# The mediating role of internalizing and externalizing symptoms in the relationship between childhood trauma and suicidality among adolescents: a structural equation model

**DOI:** 10.1186/s13034-021-00434-x

**Published:** 2021-12-23

**Authors:** Gangsan Kim, Jiyoon Shin, Jae-Won Kim

**Affiliations:** 1 Department of Psychiatry, Seoul National University Hospital, Seoul, Republic of Korea; 2 Division of Child and Adolescent Psychiatry, Department of Psychiatry, Seoul National University College of Medicine, Seoul, Republic of Korea

**Keywords:** Childhood trauma, Internalizing symptoms, Externalizing symptoms, Suicidality, Structural equation model

## Abstract

**Background:**

The objective of this study is to investigate the direct and indirect effects of childhood trauma, internalizing symptoms, and externalizing symptoms on suicidality among adolescents, thereby establishing a structural equation model.

**Methods:**

The present study uses a cross-sectional descriptive design. Among 147 adolescents aged 12–17, 93 outpatients diagnosed with major depressive disorder and 54 controls were included in the study. They completed the Early Trauma Inventory Self-Report (Short Form) and Columbia Suicidality Severity Rating Scale. Their parents completed the Child Behavior Checklist. Analyses were performed using Pearson’s correlation and structural equation modelling.

**Results:**

Childhood trauma had both direct and indirect effects, via internalizing symptoms and externalizing symptoms, on suicidality. Internalizing symptoms had a direct effect on suicidality. Meanwhile, externalizing symptoms were not directly associated with suicidality, but indirectly associated via internalizing symptoms.

**Conclusions:**

Findings provide in-depth understanding of the mediating role of internalizing symptoms and externalizing symptoms in the relationship between childhood trauma and suicidality, suggesting that the therapeutic interventions for both internalizing symptoms and externalizing symptoms may be important to prevent suicide in adolescents with childhood trauma.

**Supplementary Information:**

The online version contains supplementary material available at 10.1186/s13034-021-00434-x.

## Background

Suicidal behavior in adolescents is one of the major psychiatric problems worldwide. Since the 2000s, the suicide rate among adolescents has gradually decreased in the first decade [1, 2]. In the second decade, specifically over the past 5 years, the suicide rate among adolescents has increased in many OECD countries [3–6]. The suicide rate among Korean youth has shown no signs of decline over the past two decades. Similar to other countries, the suicide rate among Korean youth has slightly increased since 2017 [7, 8]. In Korean adolescents, the suicide rate in 2019 was 5.9 per 100,000, comprising 37.5% of the total death rate of adolescents aged 10–19 years [8]. Previous studies have reported that childhood trauma is a major risk factor for suicide-related behavior [9–12]. Childhood trauma is a stressful or traumatic experience in childhood, including physical, sexual, and emotional abuse, as well as other negative childhood experiences such as household dysfunction [13, 14]. Previous systematic reviews have reported that childhood trauma is associated with an increased risk of suicide among adolescents [15–17].

Previous studies have demonstrated that childhood trauma is associated with internalizing and externalizing disorders in adolescents [18, 19]. Experience of childhood trauma has been reportedly associated with low self-esteem, interpersonal problems, and internalizing and externalizing symptoms in adolescents [20, 21]. In one study, it was found that childhood trauma affected the central nervous system, increasing stress vulnerability, and consequently leading to the development of internalizing symptoms such as depression and anxiety among adolescents [22].

Additionally, both internalizing and externalizing symptoms in adolescents are related to suicidal risk in adolescence and early adulthood [23, 24]. Internalizing symptoms refer to inner-directed problems that cause internal psychological distress, such as anxiety, depression, somatic complaints, and withdrawal, while externalizing symptoms refer to outer-directed problems that bother other individuals and cause interpersonal conflict in the external environment, such as impulsivity, hyperactivity, delinquent, and aggressive behavior [25–27]. A study analyzed trajectories of depressive symptoms and behavioral problems, showing that both trajectories were independently associated with suicide risk [28]. Results of a systematic review noted that adolescents and young adults experiencing internalizing and externalizing symptoms are more prone to engage in suicidal behavior than those having none [29]. In contrast, some studies have shown that internalizing symptoms are associated with an increased possibility of suicide, while the other set of symptoms are not directly associated with this risk [30, 31].

Existing research has shown a significant relationship between childhood trauma and both internalizing and externalizing symptoms, and between both types of symptoms and suicidality in adolescents. Yet developmental pathways or models that explain the overall progression from childhood trauma to suicidality remain unclear. Additionally, few studies have investigated the role of both these sets of symptoms in an integrated model. Results of a longitudinal study proposed that the trajectories of anxiety and disruptiveness were mediating factors in the association between childhood trauma and suicide attempts [19]. A recent study evaluated the mediating role of comorbid symptom trajectories of internalizing and externalizing symptoms in the relationship between childhood trauma and suicide-related behaviors [32]. Although these two longitudinal studies examined the mediating effects of both internalizing and externalizing symptoms, they failed to show any direct or indirect relationships between the experience of childhood trauma and suicidality in adolescents, with either of these types of symptoms acting as mediators.

The purpose of our study was to analyze the relationships between childhood trauma, internalizing and externalizing symptoms, and suicidality among adolescents. We hypothesized that these symptoms would serve as mediators in the relationships being studied. Additionally, our study suggested a hypothetical model including developmental pathways of childhood trauma and suicidality among adolescents, using a structural equation model (SEM). We hypothesized that childhood trauma would have both direct and indirect effects on suicidality among adolescents. We also hypothesized that both internalizing and externalizing symptoms would have direct effects on suicidality among adolescents, thereby playing mediating roles between childhood trauma and risk of suicide.

## Methods

### Sample

Our sample consisted of 147 participants aged 12 to 17 years who visited the child and adolescent psychiatric outpatient clinic at the Seoul National University Hospital during August 2015–June 2018. They were a part of a study that was investigating biomarkers of antidepressant response and suicidal behavior in depressed youth (NRF-2015R1A2A2A01004501) [33, 34]. Initially, 152 subjects were recruited, but a total of 147 subjects were included in the analyses, since two depressed participants and three healthy controls withdrew their consent to participate.

The participants included 93 patients diagnosed with major depressive disorder (MDD) which was based on the Kiddie-Schedule for Affective Disorders and Schizophrenia for School-Age Children-Present and Lifetime Version (K-SADS-PL) [35, 36]. Additionally, the participants scored 40 points or higher on the Children’s Depression Rating Scale-Revised, scored 4 points or higher on the Clinical Global Impression-Severity scale, and had MDD symptoms of at least 4 weeks without psychotic features [37]. These participants had a mean age of 14.8 years (standard deviation [SD] = 1.6), and 67.7% (n = 63) of them were female.

The MDD participants, with a history of chronic medical conditions, a history of psychotic disorders, bipolar disorders, and developmental disorders, with alcohol or other substance abuse within the past 6 months, were excluded from this study. In addition, we measured the intelligence quotient (IQ) of the participants and excluded those with an IQ of less than 70. Since the original study investigated the antidepressant response, exclusion criteria were determined to prevent manic conversion due to medication [37]. Risk factors of manic conversion, such as first-degree relatives with bipolar I disorder, have been considered [38].

In addition, our study included 54 healthy control participants, recruited via flyers distributed at local schools. The recruited participants who visited our clinic were screened for psychiatric disorders using the K-SADS-PL. If a prospective participant reported receiving a diagnosis of mental illness or had any first-degree relatives with a psychiatric history, they were excluded from the study. Among the control participants, 55.6% (n = 30) were female. Mean participant age was 14.4 years (SD = 1.4).

### Measures

#### Childhood trauma

Childhood trauma was measured with 12 of the 27 items in the Early Trauma Inventory Self Report-Short Form (ETISR-SF). The ETISR-SF is a 27-item questionnaire assessing four domains of early trauma: physical, emotional, or sexual abuse, and general traumatic experiences, occurring before the age of 18. Among these domains, three were used in this study; the domain of sexual abuse was excluded. In Korea, it is difficult for adolescents to report or disclose experiences of sexual harassment due to a victim-blaming culture. For this reason, sexual abuse is likely to be underestimated. Findings related to this domain have had low reliability in previous studies [39, 40]. Each item was answered “yes” or “no” and scored dichotomously (yes = 1/no = 0) [39, 41]. We conducted confirmatory factor analysis (CFA) for the present sample, and items with factor loadings below 0.50 were excluded from the analysis. To construct the three observed variables, the items were combined and averaged. As a result, general trauma consisted of three items (with eight items excluded), physical abuse was composed of four items (with one item excluded), and five items constituted the emotional abuse domain. A previous study provided evidence for the validity and reliability of scores on the ETISR-SF in Korean adolescents [39]. In our sample, Cronbach’s α were 0.65, 0.73, and 0.82, for general trauma, physical abuse, and emotional abuse, respectively, which is evidence for good internal consistency of the instrument (see Additional file 1: Table S1).

#### Internalizing symptoms

The Korean version of the Child Behavior Checklist (CBCL) was used to measure internalizing and externalizing symptoms among the participants. The CBCL is a parent-report scale that evaluates social competence and behavior problems in children aged 6–18 years. The scale is composed of 118 behavioral problem items and 20 social competence items. We used three subscales corresponding to the internalizing broadband in CBCL, using withdrawn, anxious/depressed, and somatic subscales as observed variables to measure internalizing symptoms in the participants [42]. Each item was scored on a 3-point scale of 0 (none at all), 1 (sometimes) and 2 (often). Since item 103 was included in both the withdrawn and anxious/depressed subscales, this item was excluded from the anxious/depressed subscale, which had relatively low convergent validity, for statistical analysis. Items with factor loadings less than 0.50 were excluded from the analysis. Thus, nine items for the withdrawn subscale, 12 items for the anxious/depressed subscale, and eight items for the somatic subscale were added and averaged. The Korean CBCL has been proven to have good validity and reliability for the general child population [42]. In our study, Cronbach’s α for the withdrawn, anxious/depressed, and somatic subscales were 0.88, 0.90, and 0.85, respectively (see Additional file 1: Table S2).

#### Externalizing symptoms

Externalizing symptoms were measured with the externalizing broadband of CBCL, including delinquent subscale with 13 items and aggressive subscale with 20 items. Item 21 belonged to both subscales, so this item was excluded from the delinquent subscale, similar to that in internalizing symptoms. In statistical analysis, excluding items with factor loadings less than 0.50, 4 items (excluding eight items) were summed and averaged for the delinquent subscale, and 14 items (excluding six items) were summed and averaged for the aggressive subscale. Cronbach’s α for the delinquent and aggressive subscales were 0.70, and 0.84, respectively (see Additional file 1: Table S3).

#### Suicidality

The Columbia Suicidality Severity Rating Scale (C-SSRS) was used to measure suicidality of the participants. Composed of four constructs, this is a semi-structured interview that assesses the severity of suicidal ideation and behaviors. The four constructs are as follows: “suicidal ideation,” “intensity of ideation,” “suicidal behavior,” and “lethality of suicidal behavior.” The two former constructs evaluate suicidal ideation, while the latter two evaluate suicidal behaviors. Among them, we used “intensity of ideation (IOI)” as a measure of suicidality. The subscales that measured suicidal behaviors were excluded for two reasons. First, we utilized only continuous variables in the SEM analysis. Second, we excluded “lethality of suicidal behavior,” as this indicator had low validity and non-normal distribution in our sample. The IOI includes five items: frequency, duration, controllability, deterrents, and reasons for suicidal ideation. Items were rated on a 5-point Likert scale ranging from 1 to 5. When a participant had no suicidal ideation, all items of the participant were coded as 0. Additionally, when a participant checked the questionnaire items “does not attempt to control thought” in controllability, or “does not apply” in deterrents, and reasons constructs, these responses were coded as 0. Each item was scored on a 6-point scale ranging from 0 to 5. The convergent validity of this indicator was evaluated; the factor loadings of all items were greater than 0.50, which was acceptable. The Korean version of the C-SSRS has been proven to have good reliability and validity [43]. Cronbach’s α was 0.91 for IOI (see Additional file 1: Table S4).

### Statistical analyses

As mentioned above, we adjusted the number of measured items through item parceling to simplify the model analysis [44]. While constructing each measured variable with item parceling, some items were excluded from the statistical analysis in terms of validity [45]. Descriptive statistics were used to characterize the study sample and to test for a normal distribution. A bivariate Pearson correlation analysis was used to examine the correlation between the variables before the analysis. A preliminary CFA was performed to confirm the validity of the variables and evaluate the fit of the measurement model, thereby creating latent variables. SEM was used to analyze the fit of our hypothesized structural model and estimate the direct and indirect effects between the latent variables. To handle the missing data, the full information maximum likelihood method was used for the estimation of missing values. In addition to the original hypothesis model, two alternative models were estimated and compared to the original model. For calculating the descriptive statistics and for conducting correlation analysis, SPSS version 22.0 was used, and SEM analysis was conducted with AMOS version 26.0.

To test the hypothesized model, the model fit was analyzed by using the chi-square goodness-of-fit test and goodness-of-fit indices. The χ^2^ test compares the hypothesized model and the unconstrained model, which completely fits the data. When the probability is higher than 0.05, it can be considered that the hypothesized model fits the data. However, it is well known that since a chi-square statistic is directly affected by the sample size, especially with a large sample size, it could result in rejection of the models [46]. Thus, χ^2^/df, an indication of χ^2^ divided by degree of freedom (df), can be used to verify the fit of the model. In general, χ^2^/df values ranging from 2 to 5 are considered as acceptable [47, 48]. The root mean square error of approximation (RMSEA) is also used for model fit analysis. A value of RMSEA less than 0.08 have been considered to indicate good fit, and a value between 0.08 and 0.10 was considered to indicate moderate fit. However, recently, a cut-off value was reported to be 0.06–0.07 [48]. We also used the incremental fit indices such as the normed fit index (NFI), Tucker-Lewis index (TLI), and comparative fit index (CFI) to analyze the model fit. The values of NFI, TLI, and CFI should be higher than 0.95 for a good fit model, or higher than at least 0.90 [48–52].

Additionally, we performed a sensitivity analysis to assess the robustness of the proposed structural model. We used an independent data sample from the “validation of depression-rated scales in child and adolescent psychiatric outpatients” study (IRB No. 1908-088-1055). A total of 464 participants were recruited from the child and adolescent psychiatric outpatient clinic of Seoul National University Hospital between August 2015 and July 2019. They were aged 7 to 19 years, and 278 of whom were women. The participants responded to the same questionnaire as mentioned above in the Measure section. Among the 464 participants, 46 were excluded because they were included in the primary analysis; thus, the sample consisted of 418 participants (247 of whom were female). Among them, 196 were diagnosed with MDD, 39 with bipolar disorder, 66 with anxiety disorder, 10 with attention deficit hyperactivity disorder, 4 with conduct disorder, 2 with tic disorder, 3 with autism spectrum disorder, and 29 with eating disorder. A total of 69 participants were diagnosed with 2 different psychiatric disorders. First, the CFA model was conducted using an independent dataset. Subsequently, the structural model, which was selected as the final model in the primary analysis, was applied to the independent dataset.

## Results

### Sample characteristics

Among the 147 participants, 63.3% were female. The mean age of the participants was 14.6 years (SD = 1.6). The average IQ of the participants was 106.6 (SD = 13.1). As part of important assumptions regarding SEM, we investigated whether the data satisfied the normality assumption [53].

### Correlation analysis

Table 1 presents the correlation coefficients of the observed variables. Most of the variables were significantly and positively correlated with each other. However, the general trauma and physical abuse subscales of childhood trauma and the delinquent subscale of externalizing symptoms were not related to the observed variables that measured suicidality. Additionally, the general trauma and physical abuse subscales of childhood trauma were not associated with the somatic subscale of internalizing symptoms.

**Table 1:** Bivariate correlation analysis of the study variables

Variables	1	2	3	4	5	6	7	8	9	10	11	12	13
Childhood trauma													
1. General trauma	1												
2. Physical abuse	0.41**	1											
3. Emotional abuse	0.26**	0.52**	1										
Internalizing symptoms													
4. Withdrawn	0.18*	0.30**	0.44**	1									
5. Somatic	0.07	0.10	0.28**	0.47**	1								
6. Anxious/depressed	0.19*	0.30**	0.43**	0.81**	0.54**	1							
Externalizing symptoms													
7. Delinquent	0.44**	0.44**	0.26**	0.35**	0.24**	0.31**	1						
8. Aggressive	0.37**	0.40**	0.31**	0.60**	0.36**	0.58**	0.65**	1					
Suicidality													
9. Frequency	0.04	0.05	0.40**	0.35**	0.36**	0.42**	0.10	0.19*	1				
10. Duration	0.06	0.06	0.34**	0.37**	0.28**	0.35**	0.07	0.18*	0.82**	1			
11. Controllability	0.14	0.05	0.32**	0.34**	0.17*	0.25**	0.14	0.11	0.64**	0.72**	1		
12. Deterrents	0.10	0.06	0.29**	0.28**	0.32**	0.30**	0.15	0.17*	0.71**	0.64**	0.59**	1	
13. Reason	0.09	− 0.01	0.27**	0.41**	0.31**	0.46**	0.12	0.23**	0.75**	0.68**	0.56**	0.70**	1

^**^p < 0.01

*p < 0.05

### Preliminary CFA (test of the measurement model)

A CFA was conducted to check the model fit for the initial measurement model (see Additional file 2: Fig. S1). The χ^2^ for the initial measurement model was 143.058 (p < 0.001), and the degree of freedom for the model was 59. The fitness indices demonstrated that the model was a moderate fit. The absolute fit indices were 2.425 for χ^2^/df and 0.099 for RMSEA, and the incremental fit indices were 0.875 for NFI, 0.896 for TLI, and 0.921 for CFI. Factor loadings were higher than 0.50 for all indicators, except for the observed variable, general trauma. To improve the model fit of the initial measurement model, we excluded the variable general trauma from the latent variables of childhood trauma and conducted another CFA. The final CFA results improved the overall fit compared to the initial model (see Additional file 2: Fig. S2). χ^2^/df was 2.304 and RMSEA was 0.095, and the incremental fit indices were 0.895 for NFI, 0.916 for TLI, and 0.937 for CFI.

The construct reliabilities (CR) of the four latent variables, childhood trauma, internalizing symptoms, externalizing symptoms, and suicidality, were higher than 0.70, and the average variance extracted (AVE) was higher than 0.50 for all the latent variables, except for suicidality. For suicidality, the CR was 0.799, and the AVE was 0.445. However, even if the AVE was found to be slightly smaller than 0.50, it could be accepted if the CR is more than 0.60, so the convergent validity was considered suitable [54]. When the determination coefficient for two constructs is smaller than the AVEs of these two constructs, the discriminant validity within the construct can be confirmed. As all the determination coefficients were smaller than the corresponding AVEs, the four latent variables were shown to have good discriminant validities.

### Test of the structural model

**Fig. 1:**
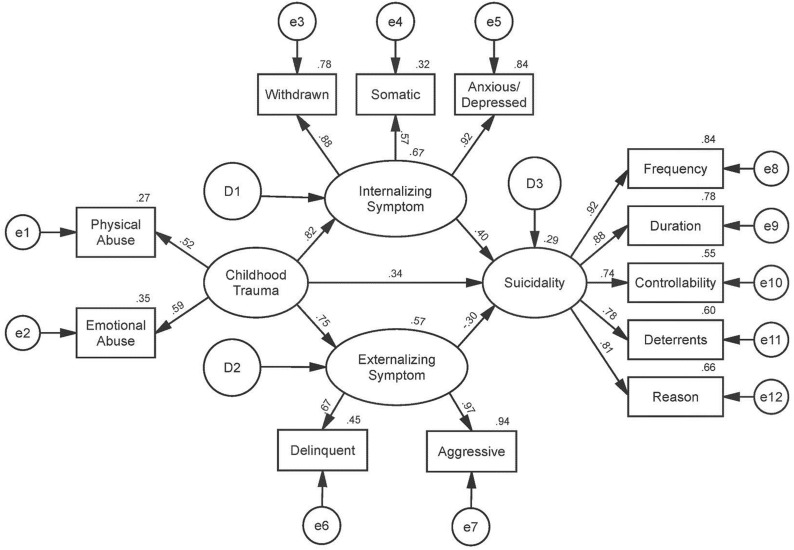
Hypothesized model. Originally hypothesized structural model. The standardized path coefficients are reported

We used SEM analysis to examine the relationships between childhood trauma, suicidality, and two other mediating variables: internalizing and externalizing symptoms. The results of the analysis of the original hypothesized model are presented in Fig. 1 and Table 2. The goodness-of-fit of the original model was analyzed, and the values of fit indices were as follows: χ^2^ = 139.225, degree of freedom = 49, and χ^2^/df = 2.841; RMSEA = 0.112; NFI = 0.873, TLI = 0.882, and CFI = 0.912. As shown in Table 2, the unstandardized (B) and standardized (β) path coefficients were estimated. In the original model, the standardized factor loadings from childhood trauma to suicidality, from internalizing symptoms to suicidality, and from externalizing symptoms and suicidality, were not statistically significant.

**Table 2:** Standardized and unstandardized path coefficients and standard errors

Path	B	S.E	Β	C.R	p-value
Original model					
Childhood trauma → internalizing symptoms	2.108	0.407	0.816	5.185	< 0.001
Childhood trauma → externalizing symptoms	1.051	0.248	0.752	4.243	< 0.001
Childhood trauma → suicidality	3.167	3.687	0.344	0.859	0.390
Internalizing symptoms → suicidality	1.413	0.897	0.397	1.576	0.115
Externalizing symptoms → suicidality	− 1.983	1.305	− 0.301	− 1.520	0.129
Competitive model I					
Childhood trauma → internalizing symptoms	0.771	0.205	0.309	3.762	< 0.001
Childhood trauma → externalizing symptoms	0.437	0.130	0.330	3.360	< 0.001
Childhood trauma → suicidality	1.958	0.839	0.222	2.335	0.020
Externalizing symptoms → internalizing symptoms	1.052	0.171	0.559	6.140	< 0.001
Internalizing symptoms → suicidality	1.750	0.442	0.495	3.964	< 0.001
Externalizing symptoms → suicidality	− 1.230	0.729	− 0.185	− 1.687	0.092
Competitive model II (final model)					
Childhood trauma → internalizing symptoms	0.776	0.206	0.311	3.767	< 0.001
Childhood trauma → externalizing symptoms	0.436	0.130	0.328	3.350	< 0.001
Childhood trauma → suicidality	2.017	0.846	0.228	2.385	0.017
Externalizing symptoms → internalizing symptoms	1.040	0.171	0.553	6.084	< 0.001
Internalizing symptoms → suicidality	1.268	0.335	0.358	3.789	< 0.001

*B* unstandardized effect, *S.E.* standard error, *β* standardized direct effect, *C.R* critical ratio

To improve the model fit, we tested a competitive model, competitive model I (see Additional file 2: Figure S3), in which a pathway from externalizing symptoms to internalizing symptoms was added to the original model. The reasons for adding this pathway to the model are as follows. In the preliminary correlation analysis, a positive correlation between internalizing symptoms and externalizing symptoms was observed. Additionally, a previous study showed that children with externalizing symptoms were more likely to develop a comorbid profile of both symptoms than children with internalizing symptoms, which suggests a causal relationship between externalizing symptoms and internalizing symptoms [55]. We added another competitive model, competitive model II, in which the non-significant pathway from externalizing symptoms to suicidality was removed from competitive model I [56]. Previous studies have suggested a non-significant relationship between externalizing symptoms and suicidality, which was supported by competitive model II [30, 31]. A comparison of the values of goodness-of-fit indices among the three models is displayed in Table 3.

**Table 3:** Model fit indices of the three structural models

Model	χ^2^	df	χ^2^/df	RMSEA	NFI	TLI	CFI
Original model	139.225	49	2.841	0.112	0.873	0.882	0.912
Competitive model I	115.201	50	2.304	0.095	0.895	0.916	0.937
Competitive model II	118.115	51	2.316	0.095	0.892	0.915	0.935

**Fig. 2:**
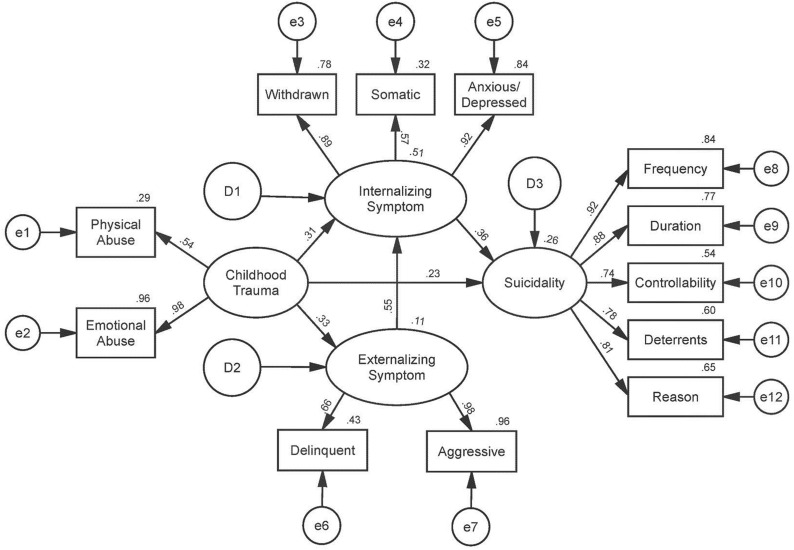
Competitive model II. Final structural model. A pathway from externalizing symptoms to internalizing symptoms is added to and the pathway from externalizing symptoms to suicidality is removed from the original model. The standardized path coefficients are reported

In terms of the model fit of the original model, RMSEA was greater than 0.10, and NFI, TLI, and CFI were less than 0.95, which does not indicate good model fit. The χ^2^ of this model was also considerably greater than the competitive models. Competitive model I and competitive model II resulted in a substantially better fit as compared to the original model. In these two models, χ^2^/df values were less than 3, the values of RMSEA were between 0.08 to and 0.10, and TLI and CFI values were higher than 0.90. As a result, competitive model I and competitive model II could be considered to be a moderate fit [46–52]. We used a chi-square difference test to compare the two competitive models. The difference between the two models was 2.914 (Δdf = 1), which was smaller than 3.84 (p = 0.05). Therefore, competitive model II was a more parsimonious model with higher degrees of freedom than competitive model I [57, 58]. For this reason, competitive model II was determined as the final model (Fig. 2).

### Direct and indirect effects of factors affecting suicidality

Standardized direct, indirect, and total effects are presented in Table 4. The significance of the indirect effects was tested by using the bootstrapping method, and all the effects between each variable were statistically significant.

**Table 4:** Standardized direct, indirect, total effects for the study variables

Independent variables	Dependent variables	Total effects	Direct effects	Indirect effects
Childhood trauma	Internalizing symptoms	0.492	0.311***	0.182**
	Externalizing symptoms	0.328	0.328***	0
	Suicidality	0.405	0.228*	0.176**
Externalizing symptoms	Internalizing symptoms	0.553	0.553***	0
	Suicidality	0.198	0	0.198**
Internalizing symptoms	Suicidality	0.358	0.358***	0

***p < 0.001

**p < 0.01

*p < 0.05

### Sensitivity analysis

A CFA was conducted on an independent data sample. Similar to the primary analysis, the observed variable of general trauma was excluded from the analysis. However, this CFA model could not be tested because the covariance matrix of latent variables was not positive definite. We also excluded withdrawn subscale of internalizing symptoms, which was expected to make the covariance matrix not positive definite, from the analysis (see Additional file 2: Fig. S4). The absolute fit indices were 1.767 for χ^2^/df and 0.043 for RMSEA. The incremental fit indices were 0.968 for NFI, 0.979 for TLI, and 0.986 for CFI. Factor loadings for all indicators were higher than 0.50, and the CR and AVE values demonstrated good construct validity for the CFA model. Next, we tested the final SEM model by applying the measurement model identified on the independent data sample (see Additional file 2: Fig. S5). The fitness indices showed that the model was a good fit. χ^2^/df and RMSEA were 2.087 and 0.051, respectively, and the incremental fit indices were 0.961 for NFI, 0.970 for TLI, and 0.979 for CFI. The unstandardized and standardized path coefficients were estimated and presented in Table 5. All the path coefficients were statistically significant.

**Table 5:** Standardized and unstandardized path coefficients on independent dataset

Path	B	S.E	Β	C.R	p-value
Childhood trauma → internalizing symptoms	0.130	0.028	0.269	4.562	< 0.001
Childhood trauma → externalizing symptoms	0.280	0.071	0.244	3.953	< 0.001
Childhood trauma → suicidality	3.074	0.485	0.436	6.336	< 0.001
Externalizing symptoms → internalizing symptoms	0.276	0.038	0.656	7.264	< 0.001
Internalizing symptoms → suicidality	1.793	0.912	0.123	1.966	0.049

*B* unstandardized effect, *S.E.* standard error, *β* standardized direct effect, *C.R* critical ratio

## Discussion

The current study was the first study that investigated the mediating role of internalizing and externalizing symptoms in the relationship between childhood trauma and suicidality among adolescents using SEM analyses. Both direct and indirect associations between childhood trauma and suicidality in adolescents were found in our study. Childhood trauma was found to be directly associated with suicidality in adolescents. Meanwhile, childhood trauma was associated with suicidality in adolescents through the following two indirect pathways. Internalizing symptoms were found to have a direct effect on suicidality in adolescents. It was observed that externalizing symptoms had no direct effect on suicidality in adolescents and indirectly affected suicidality in adolescents via internalizing symptoms.

Our finding is in line with previous studies, which demonstrated that adolescents who experienced childhood trauma are at high risk of suicide [9, 11, 12, 15–17]. Specifically, our results demonstrated that childhood trauma directly affects suicidality in adolescents without the mediating effects of internalizing symptoms or externalizing symptoms, suggesting that childhood trauma per se could be an important risk factor for suicidality in adolescents. The result that childhood trauma was indirectly associated with suicidality via internalizing symptoms is also consistent with previous studies. A study indicated that psychological stress, which was measured with depression, anxiety, and post-traumatic stress, also had a mediating role in the association between childhood trauma and suicidal ideation [59]. In this study, teenagers who experienced childhood trauma were vulnerable to internalizing symptoms, such as depression or anxiety, leading to suicidal ideation, which was in accordance with our findings.

Interestingly, externalizing symptoms did not have a direct effect on suicidality in adolescents, only having an indirect effect via internalizing symptoms. This finding corroborated previous studies that suggested that internalizing symptoms are associated with suicidal behaviors in adolescents, while externalizing symptoms are not directly associated with suicidal behaviors in adolescents [30, 31]. Aggressive or delinquent behavior in itself might not cause adolescent suffering but would contribute to their problems in other areas. Externalizing problems could prevent adolescents from getting along with family or friends, resulting in parent–child conflict or peer relationship problem [55, 60, 61]. These interpersonal conflicts and maladaptation might increase the risk of developing internalizing symptoms, such as depression and anxiety, in adolescents with externalizing problems [55]. These ideas provide evidence for adding a pathway from externalizing symptoms to internalizing symptoms to the final model of this study.

Besides, it is possible that externalizing behaviors might have protective effects on suicidal risk in adolescents. From the perspective of evolutionary psychology, externalizing behaviors of youth could be a coping strategy for adapting to a stressful environment [62, 63]. Externalizing behavior could help adolescents control their impulses for suicidal behaviors by externalizing aggression and alleviating their suffering. If that is the case, adolescent suicide would be directly increased by the internalizing symptoms and not by the externalizing ones, which corresponds well with our findings.

In fact, whether externalizing symptoms directly affect suicidality in adolescents has been a controversial issue. Some studies have reported that externalizing behaviors such as aggressive behavior and impulsivity could be a link between childhood trauma and suicidality in adolescents [64–66]. A previous study investigating the trajectory of high anxiety and high disruptiveness has reported that both trajectories independently affect the association between childhood trauma and suicide attempts [19]. These findings are inconsistent with the results of the present study. We did not directly measure impulsivity, only measuring aggressive behavior and delinquent behavior, which could be a possible cause of this inconsistency. Second, previous studies have generally measured suicidal risk by whether or not the responders had suicidal ideation or suicidal behaviors. In contrast, our study utilized the IOI subscale of C-SSRS, a semi-structured interview, resulting in a more detailed assessment of the risk and severity of suicidality [43].

Recent research has documented that comorbid trajectories of internalizing and externalizing symptoms play an important role in explaining the relationship between child maltreatment and suicidality in adolescents [32]. Similarly, we substantiated the mediating effects of both internalizing and externalizing symptoms. Furthermore, we constructed paths estimated as potentially causal relationships by integrating the findings of several studies that revealed relationships between variables [15–19, 29, 32]. We validated the fitness of a structural model that includes these paths and proposed an integrative model of developmental pathways using SEM analysis. A previous review article, which suggested a developmental model of youth suicidal behavior, is in line with our model [67]. This review article proposed that depression and aggression significantly interacted with each other, and while internalizing symptoms such as depression directly affected suicidal ideation in adolescents, externalizing symptoms such as aggression, not directly influencing suicidal ideation, had an influence on suicidal attempts of adolescents [67].

Sensitivity analyses demonstrated that our structural model had a good fit with independent clinical samples and showed that our findings were reproducible. The results of this study need to be considered in light of several limitations. First, since we used cross-sectional data, the causal relationships between variables could not be explained. Therefore, future longitudinal studies should be conducted to validate these causal relationships. Second, our sample size was 147, which was relatively small for conducting SEM analysis. When using the rule of thumb, which recommends having a sample size of at least 10 per measured variable, our sample size met the minimum requirements for SEM analysis [53]. For this reason, we could not conduct a subgroup analysis because of the small sample size. Third, our sample comprised patients with depression and a control group recruited from an outpatient clinic. Hence, the results might not be applicable to the general population of adolescents. However, inclusion of a control group could reduce the selection bias. Additionally, we performed a sensitivity analysis and showed a good fit in independent clinical data with a larger sample size. Fourth, in our sample, externalizing symptoms might have been underestimated. The sample included adolescents with depression; therefore, internalizing symptoms would be more likely to be severe than externalizing symptoms in our sample. Nevertheless, adolescents with depressive symptoms are prone to have comorbid externalizing symptoms, so that their externalizing symptoms score would be high enough for the data relating to these participants to be considered in this study [55]. Finally, to measure suicidality in the participants, we used the C-SSRS, a semi-structured interview assessing the severity of suicidal ideation and behaviors. However, only suicidal ideation was included in the analysis. Suicidal ideation is an important precursor to suicidal behavior, but not everyone with suicidal ideation acts on it [67]. Therefore, future research needs to include not only suicidal ideation but also suicidal behaviors, such as suicide attempts, in the analysis of the developmental model.

## Conclusions

The results of this study suggest that internalizing symptoms serve as a mediator of the relationship between childhood trauma and suicidality in adolescents. This finding highlights the importance of treating internalizing symptoms to prevent suicidality in adolescents with childhood trauma. Additionally, externalizing symptoms formed another pathway from childhood trauma to suicidal ideation, in which externalizing symptoms indirectly affected suicidal ideation via internalizing symptoms. These findings indicate that to prevent adolescents from committing suicide, interventions to externalizing symptoms alone might be insufficient, and it is necessary to address both externalizing and internalizing problems in an integrated way.

## Supplementary Information

**Additional file 1: Table S1.** Structure and internal consistency of childhood trauma. **Table S2.** Structure and internal consistency of internalizing symptoms. **Table S3.** Structure and internal consistency of externalizing symptoms. **Table S4.** Structure and internal consistency of suicidality.

**Additional file 2: Figure S1.** Initial measurement model (Confirmatory Factor Analysis). **Figure S2.** Final measurement model (Confirmatory Factor Analysis). **Figure S3.** Competitive model I. **Figure S4.** Measurement model on independent dataset (sensitivity analysis). **Figure S5.** Structural model on independent dataset (sensitivity analysis).

## Data Availability

The datasets used and/or analyzed during the current study are available from the corresponding author on reasonable request.

## Abbreviations

AVE: Average variance extracted
CBCL: Child-Behavior Checklist
CFA: Confirmatory factor analysis
CFI: Comparative fit index
CR: Construct reliabilities
C-SSRS: Columbia Suicidality Severity Rating Scale
df: Degree of freedom
ETISR-SF: Early Trauma Inventory Self Report-Short Form
IOI: Intensity of ideation
IQ: Intelligence quotient
K-SADS-PL: Kiddie-Schedule for Affective Disorders and Schizophrenia for School-Age Children-Present and Lifetime Version
MDD: Major depressive disorder
NFI: Normed fit index
RMSEA: Root Mean Square Approximation
SD: Standard deviation
SEM: Structural equation model
TLI: Tucker-Lewis index
