# Supplementary material for: The mediating role of internalizing and externalizing symptoms in the relationship between childhood trauma and suicidality among adolescents: a structural equation model
Source: Child Adolesc Psychiatry Ment Health. 2021 Dec 23;15:79. doi: 10.1186/s13034-021-00434-x (PMC8705103; doi:10.1186/s13034-021-00434-x)
Supplement: Supplementary file 1 — Additional file 1: Table S1. Structure and internal consistency of childhood trauma. Table S2. Structure and internal consistency of internalizing symptoms. Table S3. Structure and internal consistency of externalizing symptoms. Table S4. Structure and internal consistency of suicidality. [file 13034_2021_434_MOESM1_ESM.docx]

Additional File for

Structure and internal consistency of childhood trauma, internalizing symptoms, externalizing symptoms and suicidality

**Table S1.** Structure and internal consistency of childhood trauma.

| **Construct** | **Item** |  | **Factor loading** | **Cronbach's α** |
| --- | --- | --- | --- | --- |
| General Trauma | 5 | Separation of parents | 0.692 | 0.646 |
|  | 8 | Witnessing violence | 0.588 |  |
|  | 10 | Alcoholic parents | 0.605 |  |
| Physical Abuse | 12 | Slapped in the face | 0.663 | 0.726 |
|  | 14 | Punched or kicked | 0.616 |  |
|  | 15 | Hit with thrown object | 0.566 |  |
|  | 16 | Pushed or shoved | 0.676 |  |
| Emotional Abuse | 17 | Often put down or ridiculed | 0.752 | 0.819 |
|  | 18 | Often ignored or made to feel you didn’t count | 0.826 |  |
|  | 19 | Often told you are no good | 0.654 |  |
|  | 20 | Most of the time treated in cold or uncaring way | 0.662 |  |
|  | 21 | Parents fail to understand your needs. | 0.549 |  |
| Total |  |  |  | 0.831 |

**Table S2.** Structure and internal consistency of internalizing symptoms.

| **Construct** | **Item** |  | **Factor loading** | **Cronbach's α** |
| --- | --- | --- | --- | --- |
| Withdrawn | 42 | Would rather be alone | 0.613 | 0.879 |
|  | 65 | Refuses to talk | 0.550 |  |
|  | 69 | Secretive | 0.611 |  |
|  | 75 | Shy or timid | 0.632 |  |
|  | 80 | Stares blankly | 0.550 |  |
|  | 88 | Sulks a lot | 0.650 |  |
|  | 102 | Lacks energy | 0.768 |  |
|  | 103 | Unhappy, sad, depressed | 0.835 |  |
|  | 111 | Withdrawn | 0.739 |  |
| Somatic | 51 | Dizzy | 0.579 | 0.853 |
|  | 54 | Overtired | 0.560 |  |
|  | 56_1 | Aches, pains | 0.803 |  |
|  | 56_2 | Headaches | 0.763 |  |
|  | 56_3 | Nausea, feels sick | 0.896 |  |
|  | 56_5 | Skin problems | 0.520 |  |
|  | 56_6 | Stomach-aches | 0.771 |  |
|  | 56_7 | Vomiting | 0.608 |  |
| Anxious/depressed | 12 | Lonely | 0.594 | 0.899 |
|  | 14 | Cries a lot | 0.556 |  |
|  | 31 | Fears impulses | 0.607 |  |
|  | 32 | Needs to be perfect | 0.550 |  |
|  | 33 | Feels unloved | 0.703 |  |
|  | 35 | Feels worthless | 0.685 |  |
|  | 45 | Nervous | 0.679 |  |
|  | 50 | Too fearful or anxious | 0.733 |  |
|  | 52 | Feels too guilty | 0.598 |  |
|  | 71 | Self-conscious | 0.677 |  |
|  | 89 | Suspicious | 0.551 |  |
|  | 112 | Worries | 0.793 |  |
| Total |  |  |  | 0.939 |

**Table S3.** Structure and internal consistency of externalizing symptoms.

| **Construct** | **Item** |  | **Factor loading** | **Cronbach's α** |
| --- | --- | --- | --- | --- |
| Delinquent | 39 | Bad company | 0.522 | 0.701 |
|  | 43 | Lying or cheating | 0.549 |  |
|  | 90 | Swearing | 0.801 |  |
|  | 96 | Thinks about sex too much | 0.510 |  |
| Aggressive | 3 | Argues a lot | 0.619 | 0.844 |
|  | 16 | Mean to others | 0.614 |  |
|  | 20 | Destroys own things | 0.571 |  |
|  | 21 | Destroys other things | 0.573 |  |
|  | 22 | Disobeys at home | 0.614 |  |
|  | 27 | Jealous | 0.509 |  |
|  | 37 | Fighting | 0.611 |  |
|  | 57 | Attacks people | 0.678 |  |
|  | 68 | Screams a lot | 0.606 |  |
|  | 86 | Stubborn | 0.651 |  |
|  | 87 | Moody | 0.681 |  |
|  | 94 | Teases a lot | 0.541 |  |
|  | 97 | Threatens people | 0.601 |  |
|  | 104 | Unusually loud | 0.513 |  |
| Total |  |  |  | 0.874 |

**Table S4.** Structure and internal consistency of suicidality.

| **Construct** |  | **Factor loading** | **Cronbach's α** |
| --- | --- | --- | --- |
| Frequency | How many times have you had these thoughts? | 0.915 | 0.909 |
| Duration | When you have the thoughts, how long do they last? | 0.882 |  |
| Controllability | Could you stop thinking about killing yourself or wanting to die if you want to? | 0.740 |  |
| Deterrents | Are there things – anyone or anything – that stopped you from wanting to die or acting on thoughts of committing suicide? | 0.777 |  |
| Reason | What sort of reasons did you have for thinking about wanting to die or killing yourself? Was it to end the pain or stop the way you were feeling or was it to get attention, revenge or a reaction from others? Or both? | 0.806 |  |
