# Supplementary material for: The mediating role of internalizing and externalizing symptoms in the relationship between childhood trauma and suicidality among adolescents: a structural equation model
Source: Child Adolesc Psychiatry Ment Health. 2021 Dec 23;15:79. doi: 10.1186/s13034-021-00434-x (PMC8705103; doi:10.1186/s13034-021-00434-x)

Additional File for

Additional figures of measurement model and competitive model I

**Figure S1.** Initial measurement model (Confirmatory Factor Analysis).


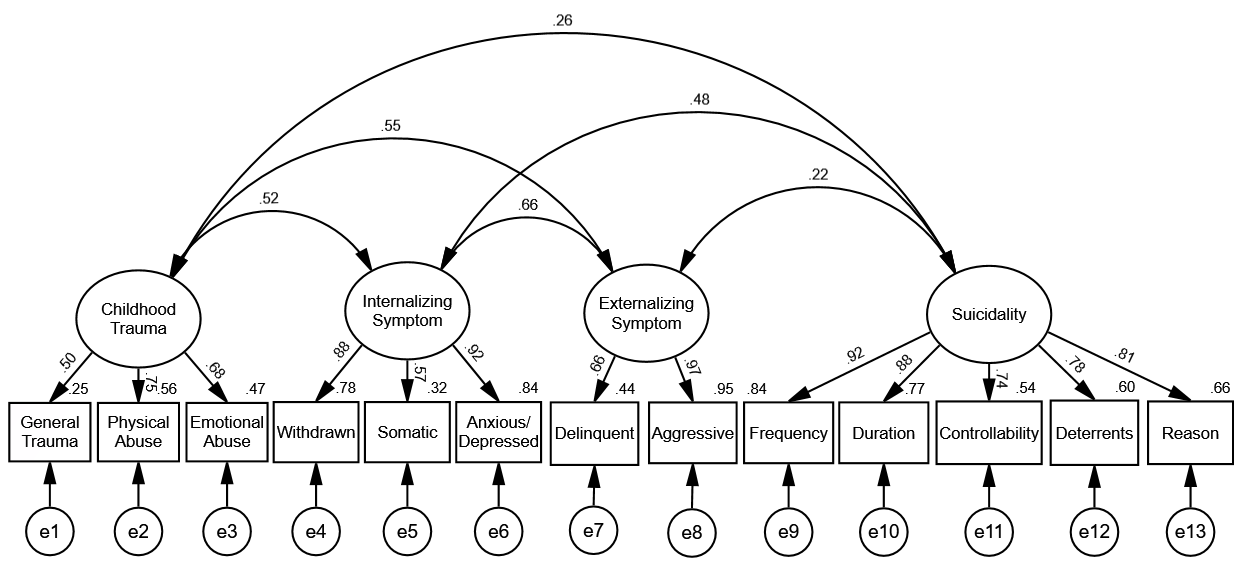


**Figure S2.** Final measurement model (Confirmatory Factor Analysis).


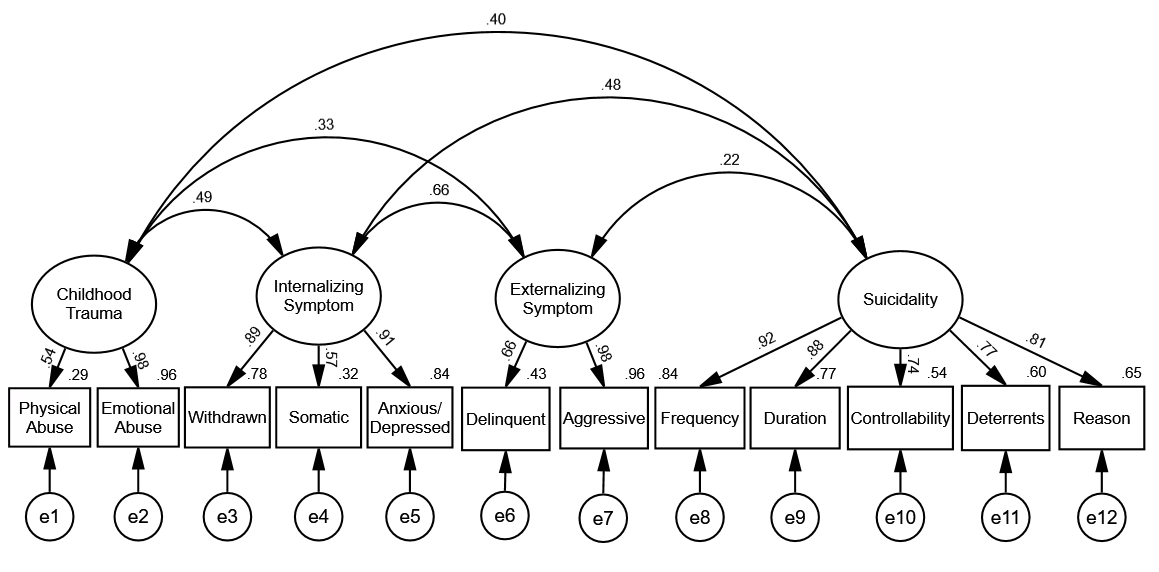


**Figure S3.** Competitive model I.


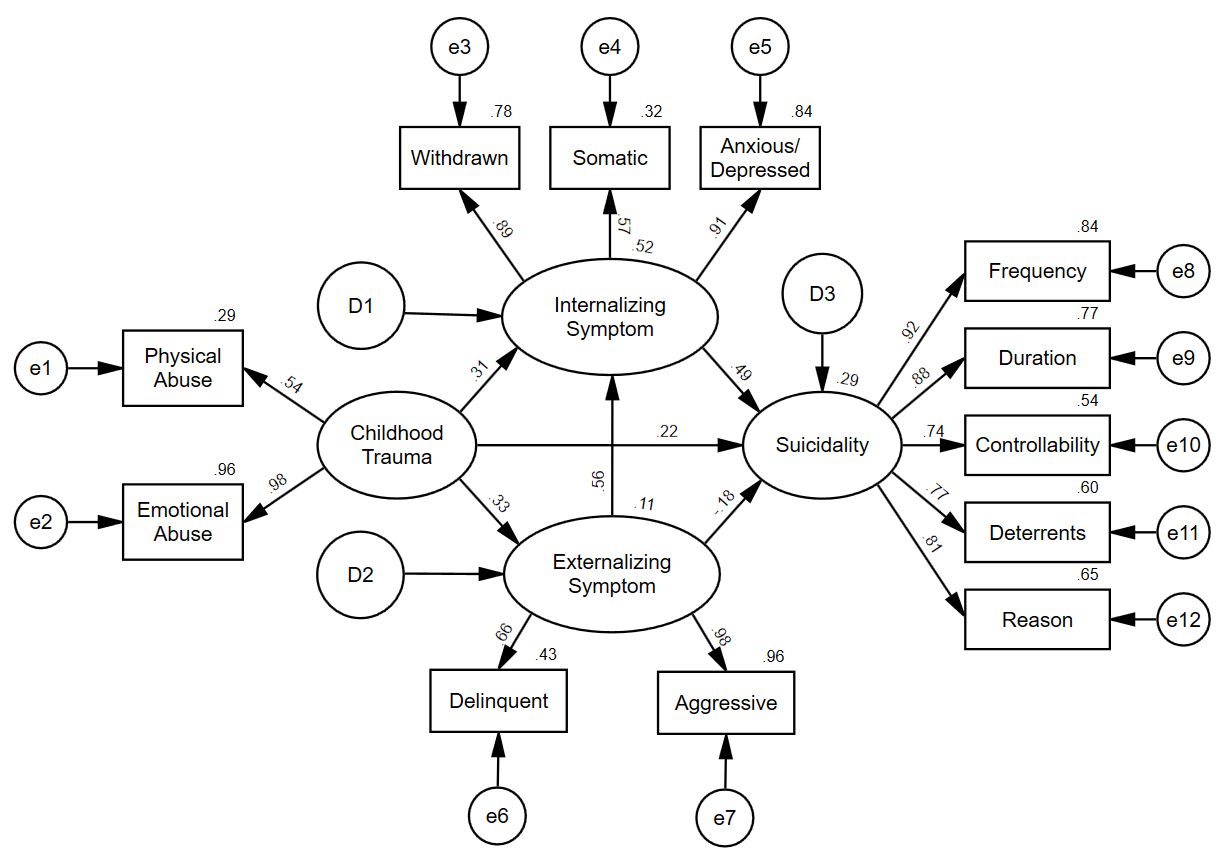


**Figure S4.** Measurement model on independent dataset (sensitivity analysis).


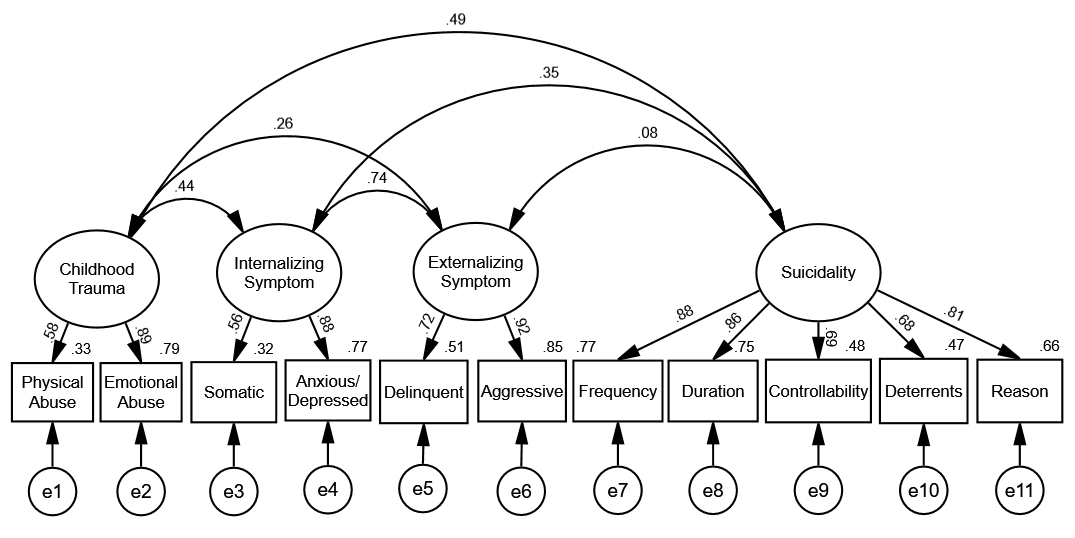


**Figure S5.** Structural model on independent dataset (sensitivity analysis).


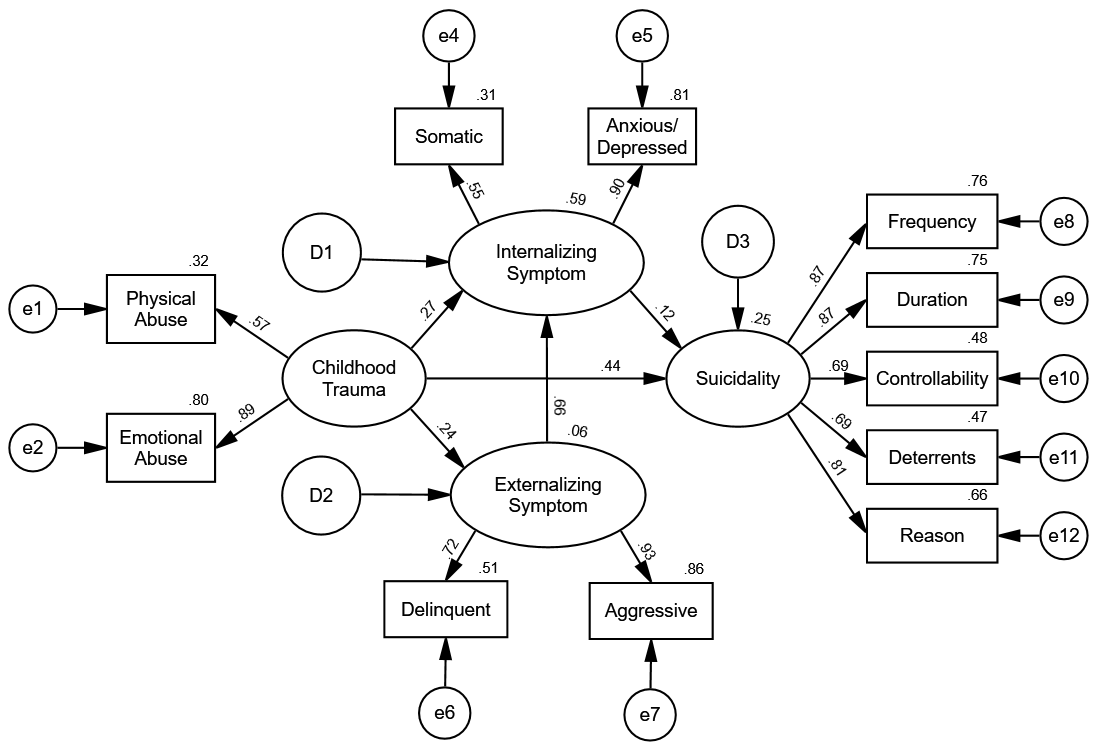

Supplement: Supplementary file 2 — Additional file 2: Figure S1. Initial measurement model (Confirmatory Factor Analysis). Figure S2. Final measurement model (Confirmatory Factor Analysis). Figure S3. Competitive model I. Figure S4. Measurement model on independent dataset (sensitivity analysis). Figure S5. Structural model on independent dataset (sensitivity analysis). [file 13034_2021_434_MOESM2_ESM.docx]
